# Cholangiopathy with Respect to Biliary Innate Immunity

**DOI:** 10.1155/2012/793569

**Published:** 2011-08-11

**Authors:** Kenichi Harada, Yasuni Nakanuma

**Affiliations:** Department of Human Pathology, Kanazawa University Graduate School of Medical Science, Kanazawa 920-8640, Japan

## Abstract

Biliary innate immunity is involved in the pathogenesis of cholangiopathies in cases of biliary disease. Cholangiocytes possess Toll-like receptors (TLRs) which recognize pathogen-associated molecular patterns (PAMPs) and play a pivotal role in the innate immune response. Tolerance to bacterial PAMPs such as lipopolysaccharides is also important to maintain homeostasis in the biliary tree, but tolerance to double-stranded RNA (dsRNA) is not found. Moreover, in primary biliary cirrhosis (PBC) and biliary atresia, biliary innate immunity is closely associated with the dysregulation of the periductal cytokine milieu and the induction of biliary apoptosis and epithelial-mesenchymal transition (EMT), forming in disease-specific cholangiopathy. Biliary innate immunity is associated with the pathogenesis of various cholangiopathies in biliary diseases as well as biliary defense systems.

## 1. Introduction

Primary biliary cirrhosis (PBC), primary sclerosing cholangitis (PSC), and hepatolithiasis in adults and biliary atresia and choledochal cyst in infants are biliary diseases in which different anatomical levels of the biliary tree are specifically affected and characterized by cholangiopathy. The biliary tree, consisting of cholangiocytes, is a system of connecting ducts that drain the bile secreted by hepatocytes into the duodenum. Cholangiocytes provide the first line of defense in the biliary system against luminal microbes originating from the intestines via portal blood and duodenum [1]. In general, although human bile is normally sterile, it can contain bacterial components such as lipopolysaccharide (LPS), lipoteichoic acid, and bacterial DNA fragments, known as pathogen-associated molecular patterns (PAMPs) [2–5], and cultivable bacteria are detectable in bile of patients with biliary diseases [1, 6–8]. Enteric bacteria, in particular, may be responsible for the chronic proliferative cholangitis associated with hepatolithiasis [1, 6]. These findings indicate that cholangiocytes are exposed to bacterial PAMPs under physiological as well as pathological conditions.

Innate immunity was initially thought to be limited to immunocompetent cells such as dendritic cells and macrophages, but epithelial cells also possess TLRs and proper innate immune systems reflecting the specific micro-environment and function of each epithelial cell type. Recent studies concerning biliary innate immunity indicate that cholangiocytes express a variety of pathogen-recognition receptors such as Toll-like receptors (TLRs) [9, 10]. Infectious agents have been implicated in the etiology or progression of cholangiopathies including cholangitis, bile duct loss, and lithiasis as a trigger or aggravating factor. Notably, several enterobacteria and viruses are speculated to be primary or secondary factors for PBC, PSC, biliary atresia, hepatolithiasis, and chronic cholecystitis [2, 3, 11–15] (Table 1). Moreover, no microorganisms showing cholangiocyte-specific tropism have been identified, suggesting that an innate immune response specific to cholangiocytes rather than PAMPs is important in the pathogenesis of cholangiopathy. This review summarizes our current understanding of the biliary innate immune system against microbial infections including the various mechanisms employed by negative regulators and their associations with the pathogenesis of cholangiopathy in biliary diseases.

## 2. Molecular Mechanisms of Biliary Innate Immunity

### 2.1. Expression of PAMP-Recognizing Receptors and Intracellular Adaptor Molecules

The TLR family are the best characterized cell surface receptors recognizing PAMPs, and 10 members (TLR1-10) have been identified in humans [16, 17]. The response to LPS is mediated by interaction with TLR4 in conjunction with the TLR4 accessory proteins MD-2 and CD14, triggering the transduction of intracellular signals followed by the activation of TLR-associated adapter proteins, myeloid differentiation factor 88 (MyD88), and IL-1 receptor-associated kinase- (IRAK-)1, leading to the activation of nuclear factor-*κ*B (NF-*κ*B) and then to the synthesis of antibiotics and proinflammatory cytokines. In contrast to bacterial PAMPs, dsRNA including viruses are recognized by TLR3, IFN-inducible helicase retinoic acid-induced protein I (RIG-I), and melanoma differentiation-associated gene-5 (MDA-5). The stimulation of these receptors by dsRNA transduces signals to activate transcription factor interferon regulatory factor 3 (IRF3) as well as NF-*κ*B. NODs (i.e., NOD1 and NOD2) are also involved in the intracellular recognition of microbes through specific interactions with derivatives of pathogen-specific peptidoglycans [18]. 

**Figure 1:**
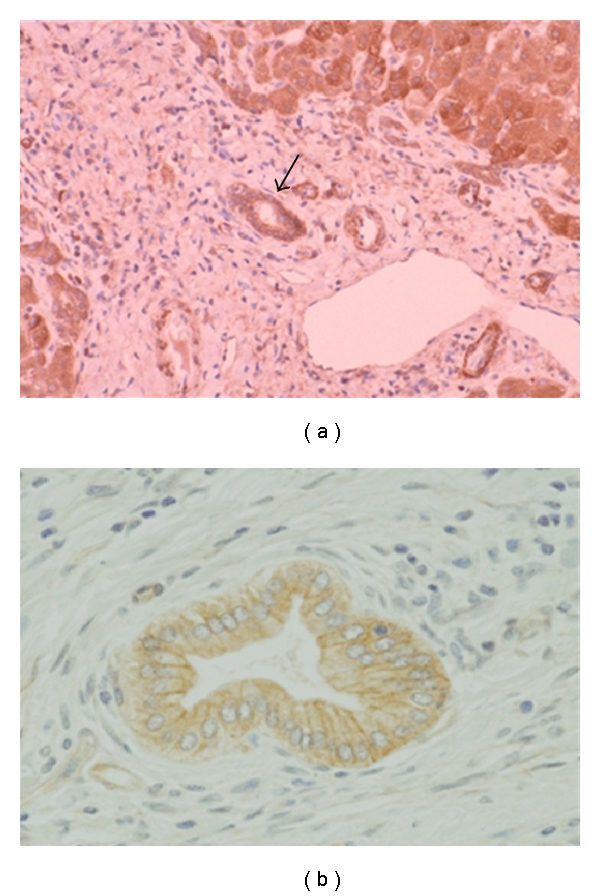
Immunohistochemistry for TLR3 in chronic hepatitis C (a) and TLR4 in primary biliary cirrhosis (PBC). The expression of TLR3 existing in endosomes is found in interlobular bile ducts (arrow in (a)) and hepatocytes in a cytoplasmic pattern. In contrast, TLR4 expression is highlighted in a membranous pattern (b).

The expression of TLRs in human and murine cholangiocytes and several human cholangiocarcinoma cell lines has been confirmed by several groups (Table 2), implicating the possible activation of biliary mucosal immunity against microbial infections [2, 19–23]. Cultured human and murine biliary epithelial cells (BECs) possess at least TLR1-TLR5, related molecules (MD-2, MyD88, and IRAK-1), RIG-I, and MDA-5 [2, 20, 23, 24]. Moreover, SV40-transformed human cholangiocytes expressed mRNAs for all ten human TLRs [19]. Immunohistochemistry has confirmed that intracellular adaptor molecules (MyD88 and IRAK-1) as well as TLRs (TLR1-TLR5) are diffusely distributed in the intrahepatic biliary tree of normal and diseased human livers, irrespective of anatomical level (Figure 1) [2, 20–22, 24, 25]. As for NODs, cultured human BECs and cholangiocytes in intrahepatic bile ducts constantly express the mRNA and protein of NOD2, but cultured BECs do not respond to the NOD2 ligand (muramyl dipeptide, MDP), indicating a suspicious functional expression (our unpublished data).

### 2.2. Recognition of PAMPs

In addition to the expression of TLRs in cholangiocytes and the biliary epithelium, the activation of TLRs has also been demonstrated during bacterial, viral, and parasitic infections. Stimulation with PAMPs including Pam3CSK4 (TLR1/2 ligand), MALP-2 (TLR2/6 ligand), peptidoglycan (TLR2 ligand), and polyinosinic-polycytidylic acid (poly(I:C), a synthetic analog of viral dsRNA, TLR3 ligand) induced the activation of NF-*κ*B, a major transcription factor downstream of TLRs, in cultured human BECs [2, 20, 23]. In addition to bacteria, *Cryptosporidium parvum *(*C. parvum*), a protozoan parasite causing intestinal and biliary diseases, also activates both TLR2 and TLR4 in cholangiocytes to initiate epithelial host responses, accompanying the recruitment of these TLRs and ganglioside GM1 to membrane rafts [26]. Membrane rafts have been implicated in TLR activation in several other cell types, including epithelial cells, following microbial infection [27]. Moreover, viral PAMPs such as double-stranded RNA (dsRNA) are also recognized by cultured BECs; cultured human BECs expressed nuclear transcription factors including NF-*κ*B and interferon regulatory factor-3 (IRF-3) on stimulation with poly(I:C), a synthetic analog of viral dsRNA [23]. These findings indicate that human BECs possess functional PAMP-recognizing receptors and an innate immune system against viruses as well as bacteria. 

In addition to microorganism components, several families of proteins originating from and produced by autocells are involved in the recognition of pathogens and the products released from injured or dying cells. In particular, endogenous factors including HMGB1, S100A8/S100A9, and heat shock proteins are known as damage-associated molecular patterns (DAMPs) [28], but a detailed analysis has not been conducted in cholangiocytes.

### 2.3. Response to PAMPs

As part of the host's defenses against infections, cholangiocytes secrete polymeric immunoglobulin A and produce several antibiotics against bacteria (lactoferrin, lysozyme, and defensins) [29–31]. Defensins, in particular, are key elements in innate immunity. Basic peptides activate against a broad spectrum of microbes including bacteria and fungus, defensins are divided into two types, *α*- and *β*-defensins. Human beta-defensins (hBDs) consisting of hBD1-hBD6 are produced by several epithelial cells including cholangiocytes and play an important role in the defense against mucosal infection. hBD1 distributes throughout the intrahepatic biliary tree and is detected in bile. Moreover, studies using cultured human BECs and SV40-transformed human cholangiocytes confirmed the constant production of hBD1 and also hBD3 [19, 22]. In contrast, hBD2 is not physiologically expressed in nondiseased livers and *de novo* expression is detected in bile ducts showing suppurative inflammation in patients with biliary diseases such as hepatolithiasis and biliary infections and also in their bile [22]. Moreover, the expression of hBD2 via the activation of NF-*κ*B occurred on stimulation by PAMPs including LPS, *E. coli, *and *C. parvum* in cultured human BECs [19, 22]. This finding suggests that hBD-1 is constantly detectable in bile samples while it plays a role in the constitutive antimicrobial defense of the hepatobiliary system and hBD2 plays a role in the localized biliary defense in cases of biliary infection. In addition to defending against bacteria, cholangiocytes possess an innate immune system to fight viral infections, because cholangiocytes have TLR3, RIG-I, and MDA-5 recognizing dsRNA viruses such as Reoviridae (reovirus and rotavirus). Stimulation with poly(I:C), a synthetic analog of viral dsRNA, induces the activation of NF-*κ*B and IRF3 and the production of key components of antiviral immunity, IFN-*β*1 and MxA [23]. In normal human liver tissues, small numbers of Kupffer cells, but no hepatocytes and cholangiocytes, exhibited MxA expression. In contrast, strong expression of the MxA protein was identified in Kupffer cells and cholangiocytes in patients with chronic liver diseases and fulminant hepatic failure [19]. These findings suggest that cholangiocytes participate directly in innate immunity and show a prompt response to pathogens without any help from immunocompetent cells such as macrophages. 

 In addition to antibiotics, cholangiocytes produce several inflammatory cytokines and chemokines such as IL-6, TNF-*α*, IL-8, fractalkine, monocyte chemotactic protein-1 (MCP-1), and CXCL16 [2, 19, 20, 32–37]. IL-6 has been demonstrated to increase DNA synthesis in human cholangiocytes *in vitro*, indicating increased proliferative activity [38]. IL-8 is closely associated with neutrophilic infiltration and its expression is found in cholangitis lenta which is usually encountered in septic patients and characterized by bile ductular proliferation, ductular cholestasis, and ductular epithelial damage [33, 39]. Chemokines produced in cholangiocytes as part of the biliary innate immune response could result in the recruitment and activation of T cells, macrophages, neutrophils, hepatic stellate cells, and NK cells to protect against biliary infection and also play an important role in bile duct-specific acquired immunity by forming periductal cytokine networks and migrating immunocompetent cells, thereby contributing to biliary mucosal defense and subsequent acquired immunity.

Cholangiocytes may also function as professional antigen-presenting cells (APCs) and contribute to the control of inflammatory reactions [40]. Cultured murine BECs constitutively expressed low levels of MHC Class I and MHC Class II molecules, and these levels were significantly enhanced by IFN-*γ* stimulation and murine cytomegalovirus (CMV) infection [41]. Moreover, murine BECs infected with murine CMV showed a progressive cytopathic effect. In contrast, in cultured human BECs, CMV-infection augmented the expression of MHC class I but not MHC class II molecules [42]. These findings suggest that CMV affects the immunogenic potential of cholangiocytes. 

TLR signals influence from fuctions of tight junctions in cholangiocytes by activating various intracellular signaling pathways. LPS disrupted the tight junctions of a rat BEC monolayer via a TLR4-dependent mechanism and LPS and *C. parvum *increased paracellular permeability by activating c-Src in rat and human BECs [43, 44]. Therefore, biliary innate immune reactions are involved in the functional regulation of tight junctions in cholangiocytes.

## 3. Regulation of Biliary Innate Immunity

TLR signaling initiates adaptive immunity which then regulates the innate immune system to maintain mucosal homeostasis. The expression of TLRs in cholangiocytes is highly regulated, but its disruption has been associated with various hepatobiliary diseases. Infecting cultured human cholangiocytes with *C. parvum *induced a significant increase in TLR4 protein, a process that appears to be associated with the production of hBD2 [19]. T cell-derived inflammatory cytokines are known to participate in the regulation of TLR expression in several cells [45, 46]. The interactions of TLRs with Th1 cytokines, in particular, participate in the pathogenesis of inflammatory bowel diseases [47]. Cholangiocytes express receptors for cytokines such as IFN-*γ*, TNF-*α*, IL-4, IL-6, and IL17, and thus, are also the target of many periductal inflammatory mediators during biliary inflammatory diseases. A Th1-type cytokine, IFN-*γ* upregulates the mRNA expression of TLR2-TLR5 and accelerates the upregulation of PAMP-induced NF-*κ*B activation in cholangiocytes, suggesting that a Th1-dominant peribiliary milieu leads to the increased susceptibility to PAMPs and the production of inflammatory cytokines and chemokines from BECs [20]. This impaired regulation of biliary innate immunity caused by the Th1-predominant milieu may be involved in the pathogenesis of cholangiopathy in biliary diseases including PBC [48]. In fact, upregulation of TLR4 and TLR9 in cholangiocytes has been reported in patients with PBC and PSC [25, 49].

Micro-RNAs play important roles in a wide range of biological events through posttranscriptional suppression of target mRNAs. Recent studies indicate that micro-RNA-mediated posttranscriptional pathways may be critical to host-cell regulatory responses to microbial infections. Cultured human BECs express let-7 family members which posttranscriptionally downregulate TLR4 expression and infections of *C. parvum *decrease the expression of let-7 resulting in the upregulation of TLR4 [50]. Moreover, microRNA-98 and let-7 suppressing cytokine-inducible Src homolog 2-containing protein (CIS, a suppressor of cytokine signaling family) at the translational level are expressed in cholangiocytes and LPS and *C. parvum* infections downregulate these mirco-RNAs, suggesting the regulation of the TLR-mediated biliary innate immune response [51].

The luminal surface of the bile duct is continually exposed to PAMPs via bile, but cholangiocytes physiologically do not elicit an inflammatory response. This lack of response to PAMPs, especially LPS, could be due to “endotoxin tolerance” and this system is important in preventing endotoxin shock in infections as well as maintaining homeostasis in organs [52]. As for negative regulatory systems of innate immunity, mechanisms compete with TLR binding and suppress intracellular TLR signaling using several molecules including extracellular soluble TLRs (sTLRs), single immunoglobulin IL-1-related protein (SIGIRR), IRAK-M (homolog of IRAK-1), MyD88s (inactive splice variant of MyD88), SARM (negative regulator of TRIF), Toll-interacting protein (Tollip), A20, SHIP (a PI3K inhibitor), and suppressor of cytokine signaling-1 (SOCS1) [52–58]. 

Our previous study using cultured BECs and cholangiocarcinoma cell lines revealed that the activation of NF-*κ*B and the increased expression of TNF-*α* caused by stimulation with PAMPs including LPS are gradually attenuated with time and that pretreatment with LPS significantly inhibits the response to subsequent stimulation, suggesting an induction of LPS (endotoxin) tolerance [59]. Moreover, pretreatment with Pam_3_CSK_4_ (TLR1/2 ligand) effectively induced tolerance to subsequent stimulation with LPS (TLR4 ligand) [52, 59]. Among several negative regulators, the expression of at least IRAK-M and Tollip has been demonstrated in human cholangiocytes and treatment with LPS and Pam_3_CSK_4_ upregulates the expression of IRAK-M, but not Tollip. IRAK-M negatively regulates TLR signaling by inhibiting the activation of IRAK-1 and MyD88 [55]. Furthermore, immunohistochemistry using human liver tissue sections confirmed that IRAK-M is diffusely expressed in intrahepatic biliary trees in both normal and diseased livers. This negatively regulated mechanism of innate immune response is important to escape hypercytokine milieu and tissue injury caused by excessive innate immune responses.

In contrast, treatment with poly(I:C), TLR3 ligand, significantly enhanced NF-*κ*B activity in fresh cultured BECs and pretreatment did not lead to tolerance to poly(I:C). [60] Levels of production of MxA and IFN-beta1 were also preserved. Therefore, TLR tolerance to a viral PAMP (poly(I:C)) is not found in BECs. Although IRAK-M mRNA expression was upregulated by stimulation with dsRNA (TLR3 ligand), no tolerance to the dsRNA was found in cultured BECs. This is reasonable because the intracellular signaling of TLR3 is a MyD88-independent pathway, that is, the dsRNA-related response is not affected by IRAK-M [17]. These findings suggest that cholangiocytes lining biliary trees are resistant to nonpathogenic commensal bacterial PAMPs, but not virus-derived dsRNA, maintaining the homeostasis of biliary innate immunity in physiological conditions. Moreover, the upregulation of IRAK-M expression on treatment with poly(I:C) is speculated to cause dsRNA-stimulated BECs to become resistant to TLR2- and TLR4-related PAMPs including LPS. Therefore, once cholangiocytes are infected by a dsRNA virus, progressive destruction caused by the biliary innate response to dsRNA and resistance to bacterial infection continues until the virus is eliminated.

## 4. Disease-Specific Cholangiopathy Associated with Biliary Innate Immunity

### 4.1. PBC

**Figure 2:**
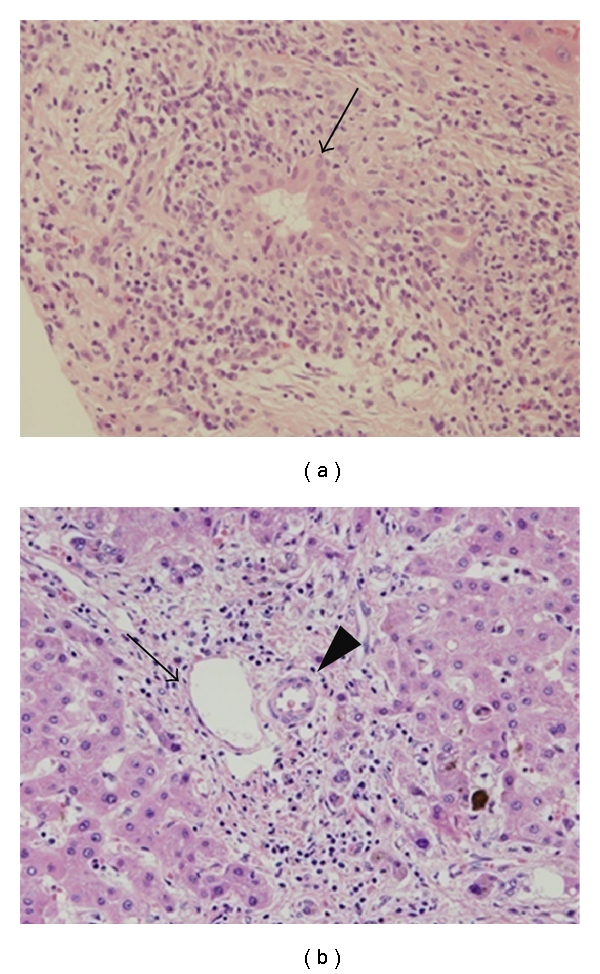
Primary biliary cirrhosis (PBC). (a) Chronic nonsuppurative destructive cholangitis (CNSDC). Damaged bile ducts (arrow) and infiltration of mixed chronic inflammatory cells surrounding bile ducts are found. (b) Bile ducts have disappeared in the portal tract. Arrowhead and arrow denote artery and portal vein, respectively.

PBC is characterized by the selective destruction and loss of interlobular bile ducts including chronic nonsuppurative destructive cholangitis (CNSDC) (Figure 2) [61]. The etiopathogenesis of PBC still remains speculative, but a high prevalence of vaginal and urinary tract infections and the presence of bacterial and viral components in bile and liver tissue and of the molecular mimicry of human and bacterial pyruvate dehydrogenase complex-E2 (PDC-E2, a major epitope of antimitochondrial antibody [AMA]) and xenobiotics are demonstrated (Table 1) [3, 5, 62–68]. Moreover, BECs translocate immunologically intact PDC-E2 to apoptotic bodies and create an apotope. The unique triad of BEC apotopes, macrophages from patients with PBC, and AMAs induces intense inflammatory cytokine production, providing a mechanism for the biliary specificity of PBC [69]. Innate immunity changes may be critical to the initiation and perpetuation of the autoimmune injury, as in the case of the enhanced response of immunocompetent cells (monocytes and memory B cells) as well as target BECs to infectious stimulation and environmental mimics [70, 71]. These findings suggest that the presence of microorganisms and the innate immune responses against them are involved in the pathogenesis, particularly cholangiopathy, of PBC. 

In PBC, the expression of TNF-*α* and IL-6 was detected in cholangiocytes from the liver of patients with PBC, suggesting the result of some biliary response including a biliary innate immune response [72]. Several studies revealed that, compared with Th2, a Th1-dominant cytokine milieu is associated with the pathogenesis including bile duct injury in PBC [48]. Cholangiocytes possess the receptor for IFN-*γ* (Th1 cytokine) and IFN-*γ* upregulates the expression of TLRs and susceptibility to PAMPs in cholangiocytes, impairing the regulation of biliary innate immunity. Moreover, IL-4 (Th2 cytokine) and IFN-*γ* up- and downregulate the expression of peroxisome proliferator-activated receptor *γ* (PPAR*γ*) showing anti-inflammatory activities in biliary innate immune response, respectively, in cultured human BECs [73, 74]. PPAR*γ* is constitutively expressed in cholangiocytes of intrahepatic small bile ducts. PPAR*γ* as well as IRAK-M, therefore, may also relate to the maintenance of biliary homeostasis as a tolerant regulator by attenuating inflammatory signals in cholangiocytes to commensal PAMPs in biles [73]. However, in PBC liver, PPAR*γ* expression is significantly downregulated in the affected bile ducts as a Th1-dominant periductal cytokine milieu [73]. Moreover, the upregulation of TLR4 and TLR9 in cholangiocytes and of TLR3 and type I IFN signaling pathways in portal tracts and parenchyma are also found in PBC [24, 25, 49]. These finding indicate an increased susceptibility to PAMPs, suggesting an association with the pathogenesis of cholangiopathy in PBC.

In addition to Th1 and Th2 cells, a third pathogenic type, Th17 cells, are involved in the pathogenesis of chronic inflammatory diseases. Human Th17 cells are characterized by the production of IL-17 (IL-17A and IL-17F) and IL-6, IL-1*β*, and IL-23 (TGF-*β* instead of IL-1*β* in mice) are critical for driving the differentiation of naïve T cells into Th17 cells and maintaining or stabilizing the functions of Th17 cells [75, 76]. In inflammatory hepatobiliary diseases including PBC, IL-17-positive mononuclear cells are scattered at the interface areas, particularly showing interface hepatitis [32]. In PBC, moreover, the periductal accumulation, particularly around cholangitis including CNSDC accompanying the expression of IL-6, IL-1*β*, and IL-23 p19, of IL-17 positive cells is found, suggesting that the Th17-related peribiliary cytokine milieu is involved in the histogenesis of the sustained cholangiopathy of PBC [32, 77]. Moreover, an *in vitro* study using cultured human BECs revealed that bacterial PAMPs (LPS and Pam3CSK4) induced the production of Th17-inducing and -maintaining cytokines (IL-6, IL-1*β*, and IL-23 p19) [32]. These results indicate that biliary innate immunity plays a role in the induction and maintenance of Th17 cells in the periductal area in cases of PBC and the differentiation into Th17 cells in periductal dendritic cells and macrophages. Th17 cells are part of the mucosal host defense system and also propagate and modulate the cholangiopathy in PBC.

Our recent study revealed that Langerin-positive Langerhans cells (LCs) are dominantly scattered around or within biliary epithelial layers of the damaged bile ducts in PBC. Moreover, experiments with cultured human BECs showed that an LC-attracting chemokine, macrophage inflammatory protein-3*α*, was produced by cholangiocytes in response to cytokines (IL-1*β*, TNF-*α*, and IL-17) and PAMPs [78]. Therefore, LCs existing around or within biliary epithelial layers are important as periductal antigen-presenting cells in PBC and the migration of LCs into bile ducts is closely associated with the periductal cytokine milieu and biliary innate immunity in PBC.

### 4.2. Biliary Atresia

**Figure 3:**
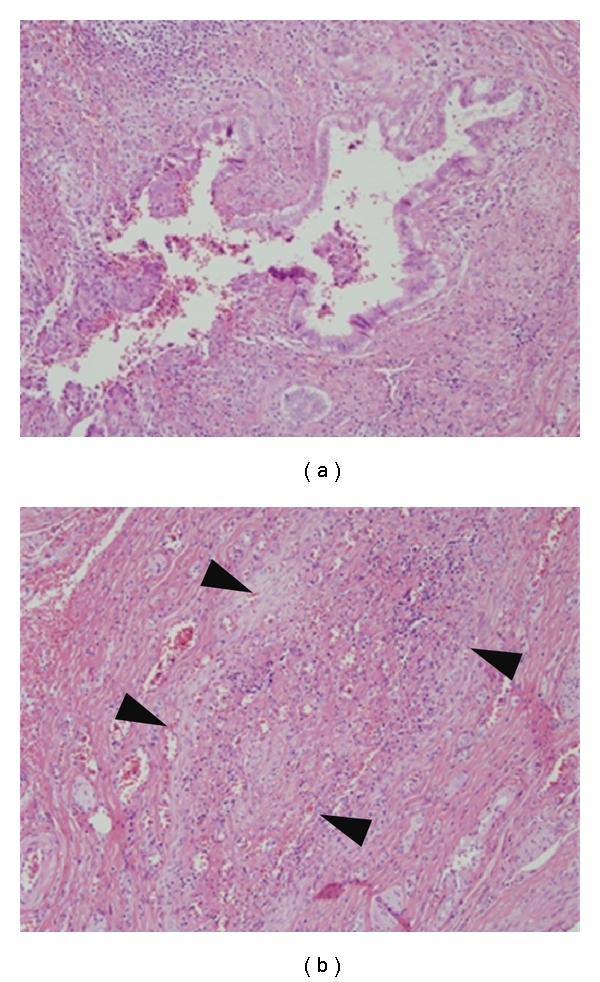
Transverse section of extrahepatic biliary remnants in biliary atresia. (a) Distorted common bile duct showing luminal occlusion with surrounding fibroplasia and inflammatory cells. (b) The common bile duct has disappeared leaving a fibrous scar (arrowheads).

Biliary atresia characterized by a progressive sclerosing obstruction of extrahepatic bile ducts (Figure 3), is a common infant biliary disease and subdivided to embryonic and perinatal types based on the clinicopathogenesis. Little is known about the etiology and pathogenesis of biliary atresia, but studies using human materials and a virus-infected rodent model suggest an association with Reoviridae (type 3 reovirus and type C rotavirus) having dsRNA, although conflicting results also have been reported [12, 79–81]. Imbalanced cell kinetics caused by enhanced apoptosis in cholangiocytes lining extrahepatic bile ducts is speculated as an important mechanism in obstructive cholangiopathy [23, 82, 83]. Human cholangiocytes are sensitive to tumor necrosis factor-related apoptosis-inducing ligand- (TRAIL-) and Fas- (CD95-)mediated apoptosis [20, 23, 84]. Moreover, because Reoviridae show epitheliotrophysm, the innate immune response against viruses is speculated to be directly associated with epithelial injury and death in biliary atresia. Our previous study demonstrated that stimulation with poly(I:C) induced the activation of NF-*κ*B and IRF-3, followed by the production of antiviral IFN-*β*1 and also enhanced apoptosis via production of TRAIL [23]. Moreover, in biliary atresia, cholangiocytes lining the remnants of extrahepatic bile ducts diffusely and constantly expressed TLR3 and showed an enhancement of TRAIL and single-stranded DNA- (ssDNA-)positive apoptosis accompanying the activation of NF-*κ*B and IRF-3 [20, 23]. A significant increase of TLR7 and antimicrobial peptide hepcidin and MxA at the mRNA and protein levels, was found in patients in the early stage of biliary atresia [85–87]. Therefore, cholangiocytes not only directly participate in the antiviral innate immune response, but also play a role in the generation of apoptotic responses to infected cells. Moreover, as described above, because the innate immune tolerance of dsRNA is lacking in cholangiocytes, the biliary damage caused by the biliary innate immune response continues until the virus disappears and directly forms the histogenesis of obstructive cholangiopathy in biliary atresia [60]. 

As the histogenesis of sclerosing lesion, the epithelial-mesenchymal transition (EMT) of cholangiocytes has been speculated to be associated with periductal fibrosis and portal fibrosis in biliary atresia [88–91]. Fundamental to EMT is a loss of normal epithelial features such as cell-to-cell adhesion molecules, the gain of mesenchymal phenotypes, and the acquisition of a fibroblast-like (spindle) morphology with cytoskeletal reorganization [92]. As mentioned above, although the biliary innate immune response to dsRNA reduces the viability of cultured human BECs via TRAIL-mediated apoptosis, the rate of cell death is approximately 70% [23]. The cells that evade apoptosis show a gradual loss of epithelial markers, CK19 (biliary-type cytokeratin in liver) and E-cadherin, and increased expression of a mesenchymal marker S100A4 (also known as fibroblast-specific protein 1) and an essential transcription factor for EMT, Snail, via increased susceptibility to transforming growth factor-*β*1 (TGF-*β*1) and the production of basic fibroblast growth factor (bFGF), demonstrating the occurrence of biliary EMT [23]. Because EMT confers resistance to apoptotic effects in fetal rat hepatocytes [93], biliary EMT is thought to be a survival mechanism and associated with an incomplete induction of apoptosis caused by the biliary innate immune response. In fact, *in vivo* studies reveal that mesenchymal markers (vimentin and S100A4) and Snail are expressed but CK19 and E-cadherin are not in cholangiocytes lining the remnants of extrahepatic bile ducts and peribiliary glands of biliary atresia [91, 94], suggesting that the occurrence of EMT in cholangiocytes is associated with an incomplete induction of apoptosis caused by the biliary innate immune response and that these surviving cells play a role in the sclerosing cholangiopathy of biliary atresia without inducing tolerance until the clearance of the virus.

## 5. Conclusion and Perspectives

Biliary innate immunity consisting of an organ-specific system is important for the mucosal immunity in intrahepatic and extrahepatic bile ducts and also associated with the pathogenesis of several cholangiopathies in biliary diseases. We speculate that biliary innate immunity is solely associated with the etiology of biliary diseases as the initial event and that the presence of causative microorganisms is not necessary in the pathogenesis of cholangiopathy caused by a subsequent acquired immunity. It is mandatory to understand the molecular basis underlying the immunophysiology and immunopathology of cholangiopathy in terms of innate as well as acquired immunity.

## Figures and Tables

**Table 1:** Bacteria and viruses speculated to be etiologic factors in biliary diseases.

Primary biliary cirrhosis
Lipopolysaccharide
Lipoteichoic acid
Helicobacter
*β*-retrovirus
Propionibacterium acnes
Escherichia coli
Mycobacterium
Novosphingobium
Lactobacillus
Chlamydia

Biliary atresia
Reovirus
Rotavirus
Cytomegalovirus
Adenovirus
Enterovirus
Ebstein-Barr virus

Primary sclerosing cholangitis
Helicobacter
*α*-hemolytic streptococcus

Hepatolithiasis
Escherichia coli
Klebsiella
Streptococcus
Pseudomonas
Bacteroides
Clostridium
Campylobacter

**Table 2:** Expression of Toll-like receptors in cultured human biliary epithelial cells (BECs), cholangiocarcinoma, and murine BECs.

	Human		Murine
	BECs	Cholangiocarcinoma	BECs

TLR1	**+ **[19]		
TLR2	**+ **[19, 20]	**+ **[2]	**+ **[2]
TLR3	**+ **[19, 20]	**+ **[2]	**+ **[2]
TLR4	**+ **[19–21]	**+ **[2]	**+ **[2]
TLR5	**+ **[19, 20]	**+ **[2]	**+ **[2]
TLR6	**+** [19, 20]		
TLR7	**+ **[19] / −*		
TLR8	**+** [19] / −*		
TLR9	**+ **[19] / −*		
TLR10	**+ **[19]		

Blanks: no reports. *Our unpublished data. Parentheses denote reference numbers.
